# A Global Analysis of Deforestation in Moist Tropical Forest Protected Areas

**DOI:** 10.1371/journal.pone.0143886

**Published:** 2015-12-03

**Authors:** B. D. Spracklen, M. Kalamandeen, D. Galbraith, E. Gloor, D. V. Spracklen

**Affiliations:** 1 School of Earth and Environment, University of Leeds, Leeds, LS2 9JT United Kingdom; 2 School of Geography, University of Leeds, Leeds, LS2 9JT United Kingdom; 3 The Rowans, Thomastown, Huntly, AB54 6AJ United Kingdom; Chinese Academy of Forestry, CHINA

## Abstract

Protected areas (PAs) have been established to conserve tropical forests, but their effectiveness at reducing deforestation is uncertain. To explore this issue, we combined high resolution data of global forest loss over the period 2000–2012 with data on PAs. For each PA we quantified forest loss within the PA, in buffer zones 1, 5, 10 and 15 km outside the PA boundary as well as a 1 km buffer within the PA boundary. We analysed 3376 tropical and subtropical moist forest PAs in 56 countries over 4 continents. We found that 73% of PAs experienced substantial deforestation pressure, with >0.1% a^−1^ forest loss in the outer 1 km buffer. Forest loss within PAs was greatest in Asia (0.25% a^−1^) compared to Africa (0.1% a^−1^), the Neotropics (0.1% a^−1^) and Australasia (Australia and Papua New Guinea; 0.03% a^−1^). We defined performance (*P*) of a PA as the ratio of forest loss in the inner 1 km buffer compared to the loss that would have occurred in the absence of the PA, calculated as the loss in the outer 1 km buffer corrected for any difference in deforestation pressure between the two buffers. To remove the potential bias due to terrain, we analysed a subset of PAs (n = 1804) where slope and elevation in inner and outer 1 km buffers were similar (within 1° and 100 m, respectively). We found 41% of PAs in this subset reduced forest loss in the inner buffer by at least 25% compared to the expected inner buffer forest loss (P<0.75). Median performance ($\tilde{P}$) of subset reserves was 0.87, meaning a reduction in forest loss within the PA of 13%. We found PAs were most effective in Australasia ($\tilde{P}=0.16$), moderately successful in the Neotropics ($\tilde{P}=0.72$) and Africa ($\tilde{P}=0.83$), but ineffective in Asia ($\tilde{P}=1$). We found many countries have PAs that give little or no protection to forest loss, particularly in parts of Asia, west Africa and central America. Across the tropics, the median effectiveness of PAs at the national level improved with gross domestic product per capita. Whilst tropical and subtropical moist forest PAs do reduce forest loss, widely varying performance suggests substantial opportunities for improved protection, particularly in Asia.

## Introduction

Tropical forests play important roles in biodiversity conservation, carbon storage and climate regulation [1]. Over the period 2000 to 2012, 1.1 million km^2^ of tropical forest were lost, with rate of forest loss increasing over this period [2]. Ongoing tropical deforestation is a significant threat to global biodiversity [3] and delivery of ecosystem services.

Protected areas (PAs) are thought to be crucial to the conservation of tropical forests [3]. Over the past few decades there has been a rapid expansion of PAs [4], which now cover >23% of moist tropical forests [5]. However, many PAs are threatened by a range of anthropogenic pressures including deforestation. The effectiveness of PAs in preventing forest loss has therefore been the subject of considerable research [5–15]. Many studies suggest PAs are at least partially effective in reducing deforestation [5, 6, 13–16], whereas others find they offer little protection [7, 17, 18]. Less is known about what makes some PAs more effective than others. How does the performance of PAs vary by country and region? Are reserves sited on more inaccessible terrain than their surroundings and to what extent does this account for any protection provided by the PA?

Many assessments of the effectiveness of PAs compare deforestation rates within the PA to those outside [3, 9, 14]. These studies have been criticised for over estimating PA effectiveness, since PAs are often located in remote regions with low deforestation rates and establishment of the PA may displace deforestation from within to outside the reserve boundary [16, 19–21]. To overcome these issues studies have assessed a wide range of covariates to correct for these biases [13, 19, 22, 23]. However, the availability of consistent data sources generally limits such analysis to regional or national level studies. Global studies have also found PAs reduce forest loss [22, 23] but limited data on covariates may not fully remove biases [23].

Here we use new global data of forest cover and forest loss [2] to make the first global assessment of forest loss within tropical and subtropical moist forest PAs using high resolution (30 m) satellite data. To quantify the effectiveness of PAs in preventing deforestation whilst addressing concerns around locational bias of PAs, we compare forest loss within inner and outer 1 km buffer zones of the PA. A number of previous studies that have used this bufferzone approach [6, 7, 9, 12] have been criticised for confounding PA effects with geographical effects [19]. To remove potential bias, our analysis corrects for any distance effects and we also analyse a subset of PAs with similar elevation and slope in inner and outer buffers. Unlike previous studies we are therefore able to quantify what fraction of PA effectiveness is down to its legal status and what is dependent on physical location. Our pan-tropical approach allows us to make a consistent analysis of PAs across the tropics, complementing studies of individual countries or regions.

## Methods

### Data

Data on global forest loss from 2000–2012 and % forest cover in 2000 was provided from a time-series analysis of Landsat 7ETM images [2] with a spatial resolution of 1 arcsecond per pixel (approximately 30 m). Definition and extent of the subtropical and tropical moist forest biome was from Nature Conservancy [24]. Data on PA location, boundaries and year of establishment are from the World Database on Protected Areas [25]. Data on slope was derived from the CSI-CGIAR version [26] of the 3 arc-second resolution SRTM digital elevation model from NASA [27].

For information on the effectiveness of national governance (Control of Corruption and Political Stability) we used the Worldwide Governance Indicators [28] from 2006. Information on Foreign Debt (% GDP), Gross Domestic Product (GDP) per capita, road density and rural population were also taken from [28].

### Analysis

Quantifying the effectiveness of PAs through direct comparison of forest loss rates within and outside PAs has faced criticism [16, 19–21]. Two issues have been identified. Firstly, the location of PAs are often biased to remote and inaccessible areas with less danger of deforestation [21]. Secondly, deforestation in regions surrounding the PA is potentially modified by the presence of the PA itself [16]. To overcome these issues previous studies have assessed a wide range of additional covariates and used matching techniques in an attempt to remove bias [13, 19, 22, 23]. These methods require extensive data on various covariates that is not consistently available at the pan-tropical scale. Some global studies have used a more limited set of variables in an attempt to remove bias [22, 23]. However, it is difficult to assess whether these approaches adequately remove bias [23].

We argue that a careful buffer zone analysis is an appropriate option for a pan-tropical study covering a wide range of countries. We compare forest loss rates in multiple buffers including an inner 1 km buffer within the PA adjacent to an outer 1 km buffer directly outside the PA. Gradual environmental gradients such as distance to roads and cities cannot account for any sizeable difference in deforestation in adjacent buffers (which are essentially the same location). To ensure we remove any difference in forest loss rates between the inner and outer buffers that is due to differences in deforestation pressure, we correct using the rate of forest loss with distance from the PA boundary (explained in detail below). Strong environmental gradients may occur between the inside and outer 1 km buffers, giving the PA a degree of physical protection unrelated to its legal status [23]. For example, a PA may be located on steeper and higher elevations with a lower risk of deforestation. We investigated this possibility by computing the mean elevation and slope in the inner and adjacent outer buffer zones for all reserves. We then analysed forest loss within the subset of PAs where slope and elevation in the inner and outer buffers was similar. To explore the impact of the PA on surrounding forest loss, we analysed forest loss within concentric buffers out to a distance of 15 km from the PA boundary. This analysis allows us to demonstrate that PAs are not displacing deforestation to the forest immediately outside the reserve.

We calculated forest loss (*r*) as the fraction of forest cover present in 2000 (*F*_2000_) that had experienced forest loss (L) by 2012 (*r* = *L*/*F*_2000_). We defined forest as areas with more than 10% tree canopy cover [29]. For each PA analysed, we calculated forest loss within a) the entire PA (*r*_*PA*_), b) an inner buffer of width 1 km (*r*_*in*_), that is the part of the PA within 1 km of the PA boundary, as well as 7 buffer zones outside the PA boundary: c) five 1 km wide bufferzones outside the PA boundary: 0–1, 1–2, 2–3, 3–4 and 4–5 km from the boundary (*r*_*out*_, *r*_2_, *r*_3_, *r*_4_ and *r*_5_) and e) two 5 km wide bufferzones: 5–10 and 10–15 km outside the PA boundary (*r*_10_ and *r*_15_). Average forest loss between 1 km and 5 km from the PA boundary is also reported (*r*_1|5_). Outer buffers only included non-protected land—any part of the buffer that lay within another PA was not included in the analysis. For a more accurate comparison, the inner buffer consisted only of that portion of PA adjacent to the outer 1 km buffer.

A simple definition of a PA’s performance (*P’*) is the ratio of forest loss in the inner buffer (*r*_*in*_) to the forest loss in the 1 km outer buffer (*r*_*out*_): *P*′ = *r*_*in*_/*r*_*out*_. However, deforestation pressure typically declines with distance from roads and settlements, meaning *r*_*in*_ would be slightly lower than *r*_*out*_, even in the absence of the protected area. This effect means that use of *r*_*in*_ and *r*_*out*_ would lead to a slight overestimate of reserve effectiveness. To account for this we calculate the gradient (G) of deforestation rate with respect to distance from the PA boundary (between *r*_*out*_ and *r*_10_). We use this gradient to estimate the expected rate of deforestation in the inner buffer that would have occurred in the absence of the reserve (*r*_*inex*_ = *r*_*out*_-*G*). We then calculate performance as: *P* = *r*_*in*_/*r*_*inex*_. Correcting for this effect only slightly modifies *P*, with changes of 1–3% dependent on the country.

A value of *P<1* means the deforestation rate in the inner buffer is lower than would be expected in the absence of the reserve (*r*_*in*_ < *r*_*inex*_). Values of P > 1 occur where inner buffer forest loss is greater than expected (*r*_*in*_ > *r*_*inex*_). In practice the short 1km distance between these buffers means that the correction (gradient G) is small and *r*_*inex*_ ≈ *r*_*out*_. In reserves with very little deforestation pressure (low *r*_*out*_), *P* may be > 1 simply because there is little deforestation to prevent. However, we found that very few PAs had *r*_*out*_ < 0.01%*a* ^−1^ (n = 154, <5% of all PAs) indicating this was not an issue in our analysis. We focus our analysis on median *P* ($\tilde{P}$), which is less influenced by the few PAs with very low *r*_*out*_.

Calculating reserve effectiveness through the ratio of fractional forest loss (*P* ∝ [*F*_*out*_/*F*_*in*_]) may also overestimate reserve effectiveness. Where the outer buffer has a lower forest cover compared to the inner buffer (across all reserves median treecover is 93% in inner buffer compared to 85% in the outer), the same absolute area of forest lost in both buffers would result in P<1. To explore the potential size of this effect, we also calculated PA effectiveness based on absolute forest loss in the inner and outer buffers (*P*_*abs*_ = *L*_*in*_/*L*_*out*_). Across all reserves, this method changes P by less than 3% compared to the standard method and so is not a major source of error in our analysis.

PAs that were completely surrounded by other reserves, had *F*_2000_ < 5%, were entirely surrounded by water or lay largely outside the moist tropical forest biome were discarded. Due to the availability of forest loss data (2000 to 2012), our analysis is restricted to PAs that have been established pre-2000. This is a limitation especially in regions where there has been a large expansion of the PA network over this period [13, 14, 30]. Finally, only countries with at least 3 eligible PAs were considered.

With these restrictions we analysed a total of 3376 tropical and subtropical moist forest PAs across 56 countries: 20 in Africa (550 reserves), 14 in Asia (1626 reserves), 20 in the Neotropics (1134 reserves) and 2 in Australasia (66 reserves). We classed Indonesia as an Asian and Papua New Guinea as an Australasian country. All the PAs analysed were located between 34°*S* and 31°*N*. The total area of the reserves analysed was over 2.2 million km^2^, representing slightly over 10% of the moist tropical forest biome. The full dataset for all reserves can be seen in S1 Dataset.

We performed multiple linear regression analysis to explore the relationship between national level median performance ($\tilde{P}$) and 8 national level variables (Rural population growth, rural population density, GDP/capita, foreign debt (as % GDP), control of corruption, political stability and absence of violence/terrorism, density of roads (ratio of length of country’s total road network to country’s land area), fractional forest area). These variables were checked for multicollinearity (Variance Inflation Factor <2.5) and the model fitted using the best subsets model selection method with the best models selected based on the lowest AIC (Akaike Information Criterion) values.

Our evaluation of effectiveness of PAs was restricted to forest loss; we did not assess the role of PAs in preventing poaching, selective timber harvesting or grazing.

## Results

Table 1 reports forest loss within moist tropical forest PAs (r_*PA*_) at the continental level over the period 2000 to 2012. Median *r*_*PA*_ (${\tilde{r}}_{PA}$) was greatest in Asia (0.16% a^−1^) and Africa (0.15% a^−1^), less in the Neotropics (0.09% a^−1^) and very low in Australasia (0.002% a^−1^). At the pan-tropical scale, ${\tilde{r}}_{PA}$ (0.12% a^−1^) was less than mean *r*_*PA*_ (0.4% a^−1^), indicating a small number of reserves with rapid forest loss. When weighted by reserve area, mean *r*_*PA*_ was 0.13% a^−1^, suggesting that larger reserves typically have lower fractional rates of forest loss. Scharlemann et al. [31] also found 0.13% a^−1^ forest loss within PAs in the humid tropics over the period 2000 to 2005. In our analysis, PA forest loss (weighted by reserve area) was greatest in Asia (0.25% a^−1^), and less in Africa (0.1% a^−1^), Neotropics (0.1% a^−1^) and Australasia (0.03% a^−1^). We found that 39% of PAs experienced *r*_*PA*_ greater than 0.2% a^−1^, compared to 52.5% of PAs in a meta-analysis of previous studies [32].

**Table 1:** Intercontinental comparison of reserves’ deforestation rates and performance. Variables included are: mean area of PA (A), median forest loss within PA ($\tilde{r}$), area weighted forest loss within PA (d), median forest loss in the 1 km outside buffer (${\tilde{r}}_{out}$), fraction of PAs with *r*_*out*_ > 0.1%*a* ^−1^ (*F*), mean performance ($\bar{P}$, *P* = *r*_*in*_/*r*_*inex*_), median performance ($\tilde{P}$), fraction of PAs that are effective defined as *P* < 0.75(*E*) and highly effective (H) *P* < 0.5. Values are shown for all PAs and for the subset of PAs with similar slope and elevation in inner and outer 1 km buffers.

	A (km^2^)	$\tilde{r}\left(\%{\text{a}}^{-1}\right)$	$d(\%a^{-1})$	${\tilde{r}}_{out}(\%a^{-1})$	F (%)	$\bar{P}$	$\tilde{P}$	*E*|*H* (%)
All PAs								
**Africa (n = 550)**	**500**	**0.15**	**0.10**	**0.28**	**78**	**1.09**	**0.78**	**48|29**
**Asia (n = 1626)**	**270**	**0.16**	**0.25**	**0.26**	**72**	**1.03**	**0.87**	**42|27**
**Australasia (n = 66)**	**93**	**0.002**	**0.03**	**0.08**	**42**	**0.42**	**0.07**	**76|71**
**Neotropics (n = 1134)**	**1310**	**0.09**	**0.10**	**0.25**	**73**	**0.82**	**0.64**	**57|42**
PAs with buffers of similar slope and elevation								
Africa (n = 341)	645	0.18	0.11	0.32	84	1.04	0.83	43|26
Asia (n = 826)	301	0.23	0.38	0.31	73	1.2	1	31|18
Australasia (n = 25)	220	0.025	0.03	0.13	56	0.48	0.16	68|64
Neotropics (n = 612)	1997	0.11	0.16	0.31	75	0.91	0.72	52|39

**Fig 1:**
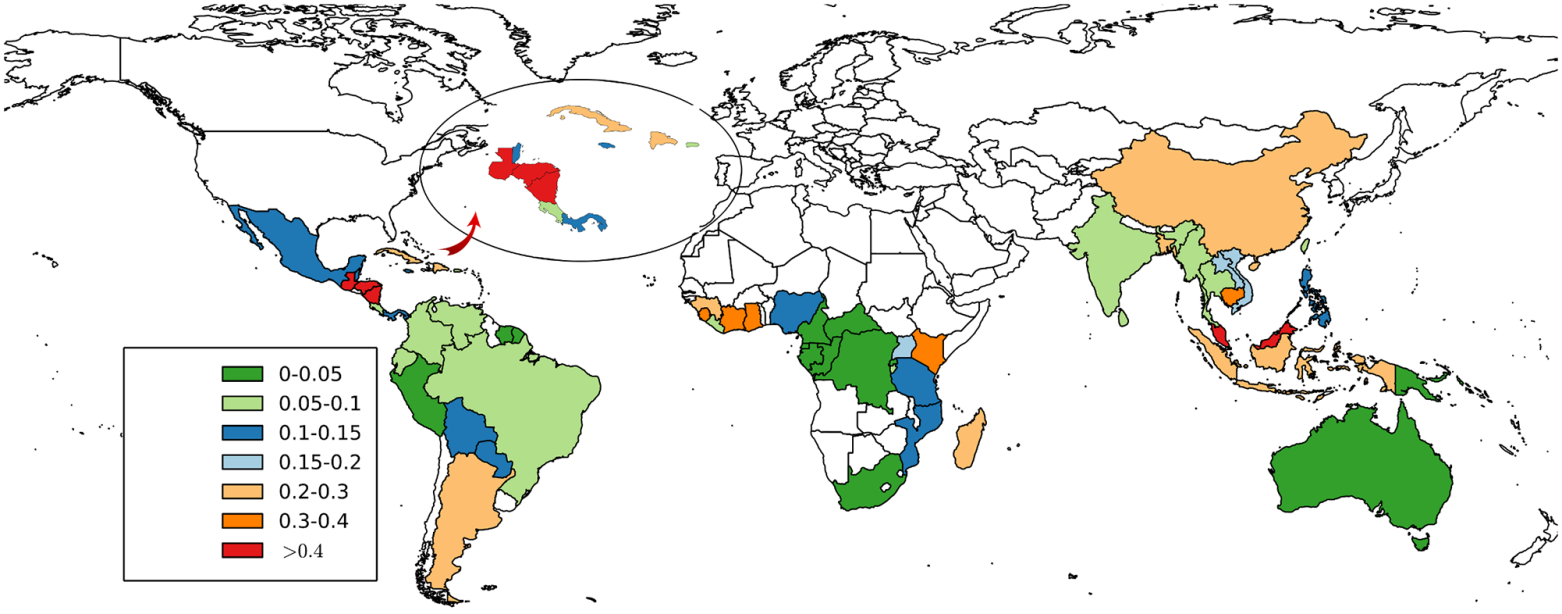
Area-weighted PA deforestation from 2000–2012 across all 56 countries. National mean annual forest loss (%*a* ^−1^) within tropical and subtropical moist forest PAs (weighted by reserve area) over the period 2000 to 2012.

Fig 1 shows the annual rate of forest loss within PAs at the national level. Countries with large and relatively remote PAs in the Congo and the Amazon have low deforestation rates (<0.1% a^−1^). Higher deforestation rates (> 0.3% a^−1^) within PAs are seen across many countries in Asia, central America and west Africa. The lowest deforestation rate was in Cameroon (0.005% a^−1^) and the highest in Nicaragua (1% a^−1^) and Malaysia (0.8% a^−1^).

**Fig 2:**
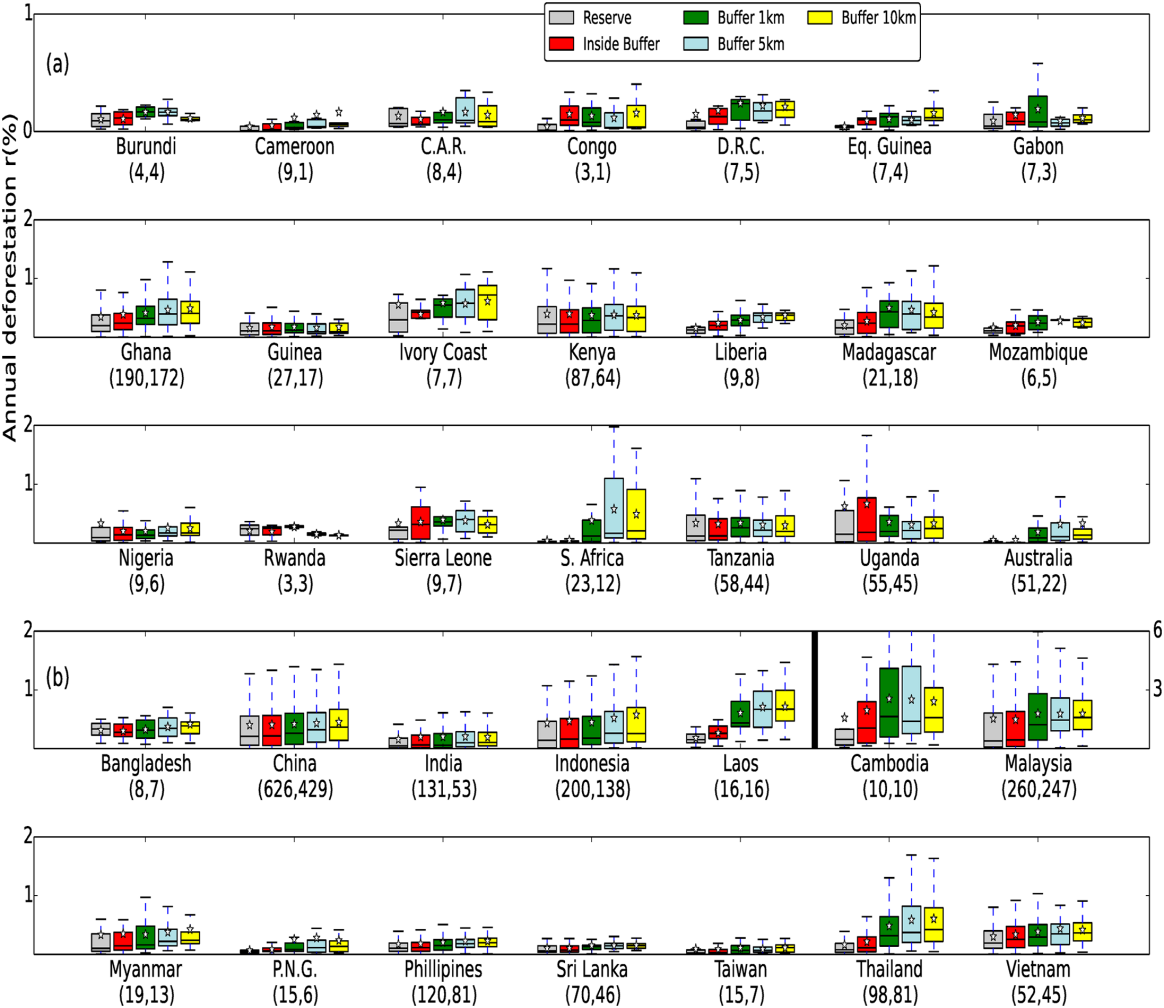
PA Deforestation rates for African, Asian and Australasian countries. Annual % forest loss (*r*) within PAs and buffers *r*_*in*_, *r*_*out*_, *r*_1|5_ and *r*_10_ for (a) African countries + Australia, (b) Asian countries + Papua New Guinea. Note the different axes for Cambodia and Malaysia. Numbers in brackets show the number of analysed PAs and the number of PAs experiencing substantial deforestation pressure (*r*_*out*_> 0.1% a^−1^). Boxes show 25th and 75th percentiles, whiskers show the first quartile -1.5*interquartile range(IQR) and third quartile +1.5*IQR, stars: mean, line: median).

**Fig 3:**
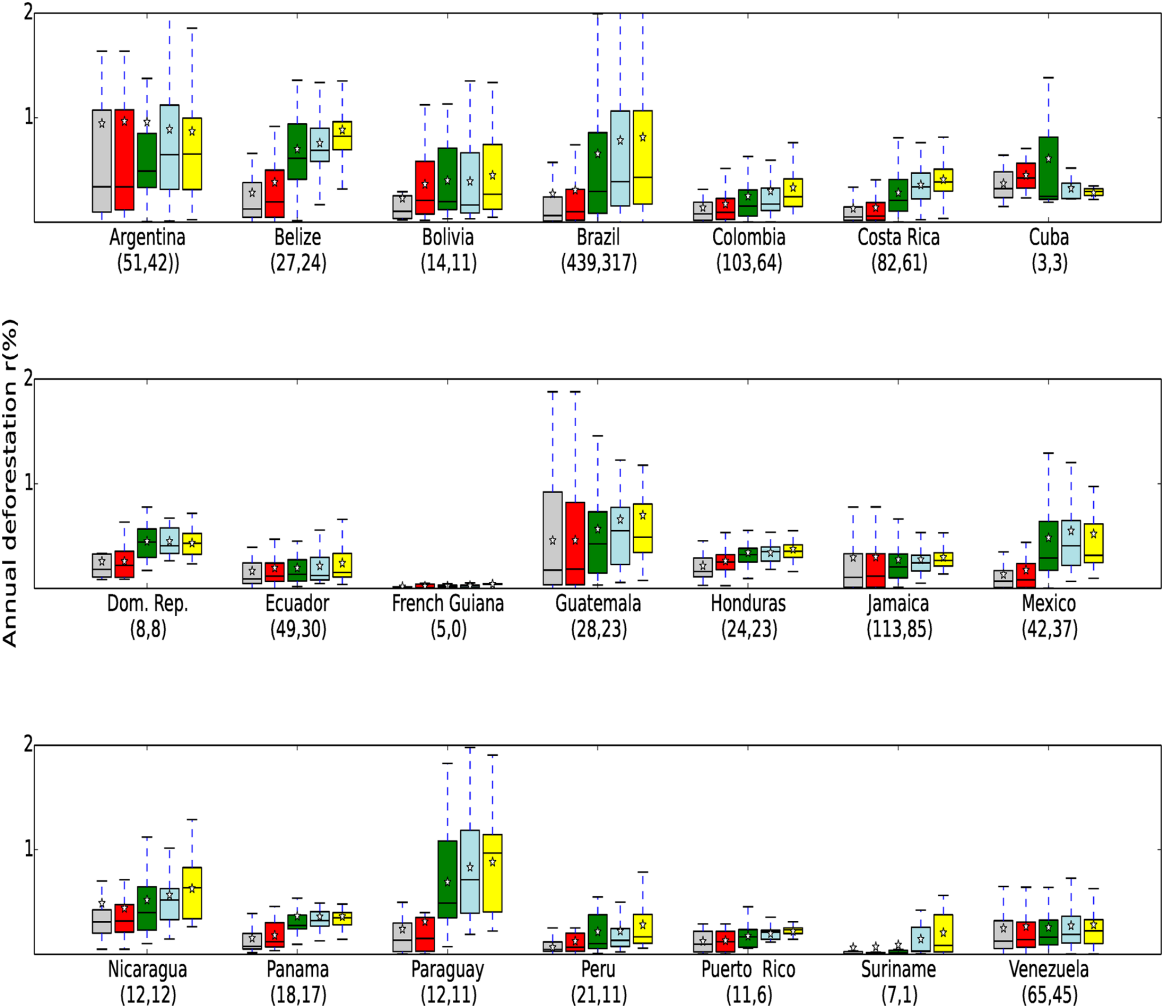
PA Deforestation rates for Neotropical countries. Annual % forest loss (*r*) within PAs and buffers *r*_*in*_, *r*_*out*_, *r*_1|5_ and *r*_10_ for Neotropical countries. See Fig 2 for details.

Figs 2 and 3 show annual forest loss rates (*r*) within PAs and the inner buffer (*r*_*out*_) compared to 3 outer buffers (*r*_*out*_, aggregate bufferzone *r*_1|5_ and *r*_10_) at the national level. Nearly three quarters (73%) of all PAs were experiencing substantial deforestation pressure (*r*_*out*_ > 0.1% a^−1^), with little difference between Africa (78%), Asia (72%) and the Neotropics (73%), though considerably lower in Australasia (42%) (Table 1). Less than 15% of PAs in French Guiana, Cameroon and Suriname experienced substantial deforestation pressure. Many countries had more than 90% of their PAs experiencing substantial deforestation pressure, with the greatest forest loss in the outer buffers occurring in Cambodia and Malaysia where ${\tilde{r}}_{out}$ exceeded 0.8% a^−1^.

**Fig 4:**
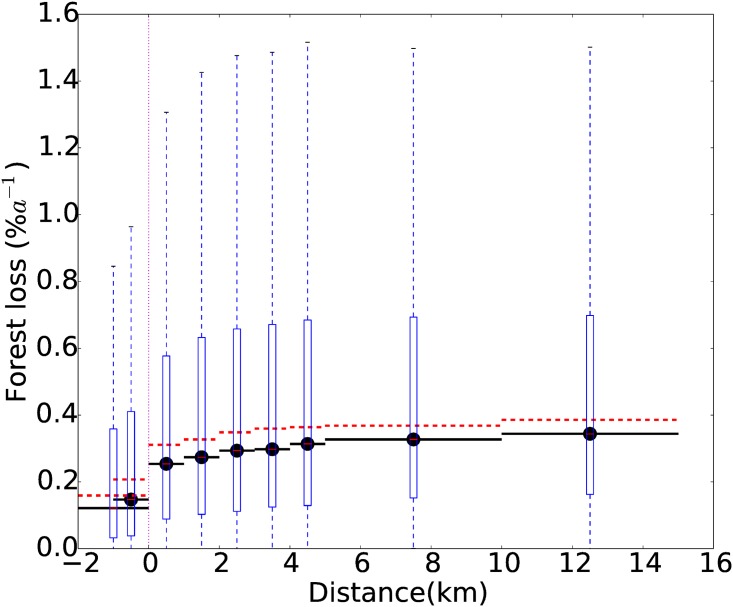
Forest loss rates (*r*) as a function of distance from PA boundary. Horizontal lines indicate the distance over-which *r* is calculated. Median forest loss is shown for all PAs (black line) and the subset of reserves with similar slope and elevation within inner and outer 1km buffers (red dotted line). Vertical dotted line indicates the PA boundary. Box and whiskers are shown for all PAs and are as Fig 2. Boxplot at Distance = -1km represents forest loss rates in PA.

There is a likelihood that the presence of a reserve impacts on the deforestation rates of the surrounding non-protected land. This “spillover effect” is an obvious critique of our bufferzone method. The effect of a reserve on the deforestation of its immediate surroundings is of interest in its own right—a PA is not so useful if its protection is at the expense of its surroundings. Therefore, we examine how the median forest loss changes between inside the PA (${\tilde{r}}_{PA}$), the inner 1 km buffer (${\tilde{r}}_{in}$) and the outer buffers (Fig 4). Median forest loss in the outer buffers (0.25—0.34% a^−1^) is faster than the median forest loss rate inside PAs (0.125% a^−1^). Deforestation rate declines as the PA boundary is approached, but the largest difference in forest loss rate occurs across the PA boundary between ${\tilde{r}}_{in}$ (0.15% a^−1^) and ${\tilde{r}}_{out}$ (0.25% a^−1^). We compared the difference in forest loss rates between the different buffers as a function of horizontal distance from the PA boundary. The difference in median forest loss between ${\tilde{r}}_{in}$ and ${\tilde{r}}_{out}$ (1 km apart) is 0.1% a^−1^ km^−1^, substantially greater than the difference between ${\tilde{r}}_{out}$ and ${\tilde{r}}_{5}$ (0.015% a^−1^ km^−1^) or ${\tilde{r}}_{10}$ and ${\tilde{r}}_{15}$ (0.003% a^−1^ km^−1^). The small reduction in forest loss rates between ${\tilde{r}}_{15}$ and ${\tilde{r}}_{out}$ is likely due to a decline in deforestation pressure due to remoteness. The colocation of the largest change in forest loss rate with the PA boundary suggests the presence of the PA is the cause of the reduced forest loss rates. If the PA displaced deforestation to the immediate surroundings, faster forest loss would occur in the buffer directly outside the PA compared to the more distant buffers. We find the opposite is true, with slower rates of forest loss close to the PA boundary (${\tilde{r}}_{out}$) compared to 1–5, 5–10 and 10–15 km buffers, suggesting that the presence of a reserve does not encourage the deforestation of its immediate vicinity. Note, however, that the reserves in our analysis could still be displacing deforestation further afield—potentially to different countries or even continents not included in our study.

Before analysing the effectiveness of PAs, we first explored the potential for differences in terrain (slope and elevation) between the inside and outside of the PAs to alter forest loss rates. We calculated mean slope (*S*) and elevation (*E*) in the inner 1 km and outer 1 km buffers of each PA. At the pan-tropical scale, PAs were located on steeper terrain with higher elevation compared to their surroundings, with median *S* (*E*) for the outer 1 km buffer of 8.4° (364 m) compared to a median *S* of 9.4° (396 m) in the inner 1 km buffer. Higher elevation and steeper slopes within PAs has been found previously [22], though note that we compare the inner 1 km buffer rather than the entire PA.

**Fig 5:**
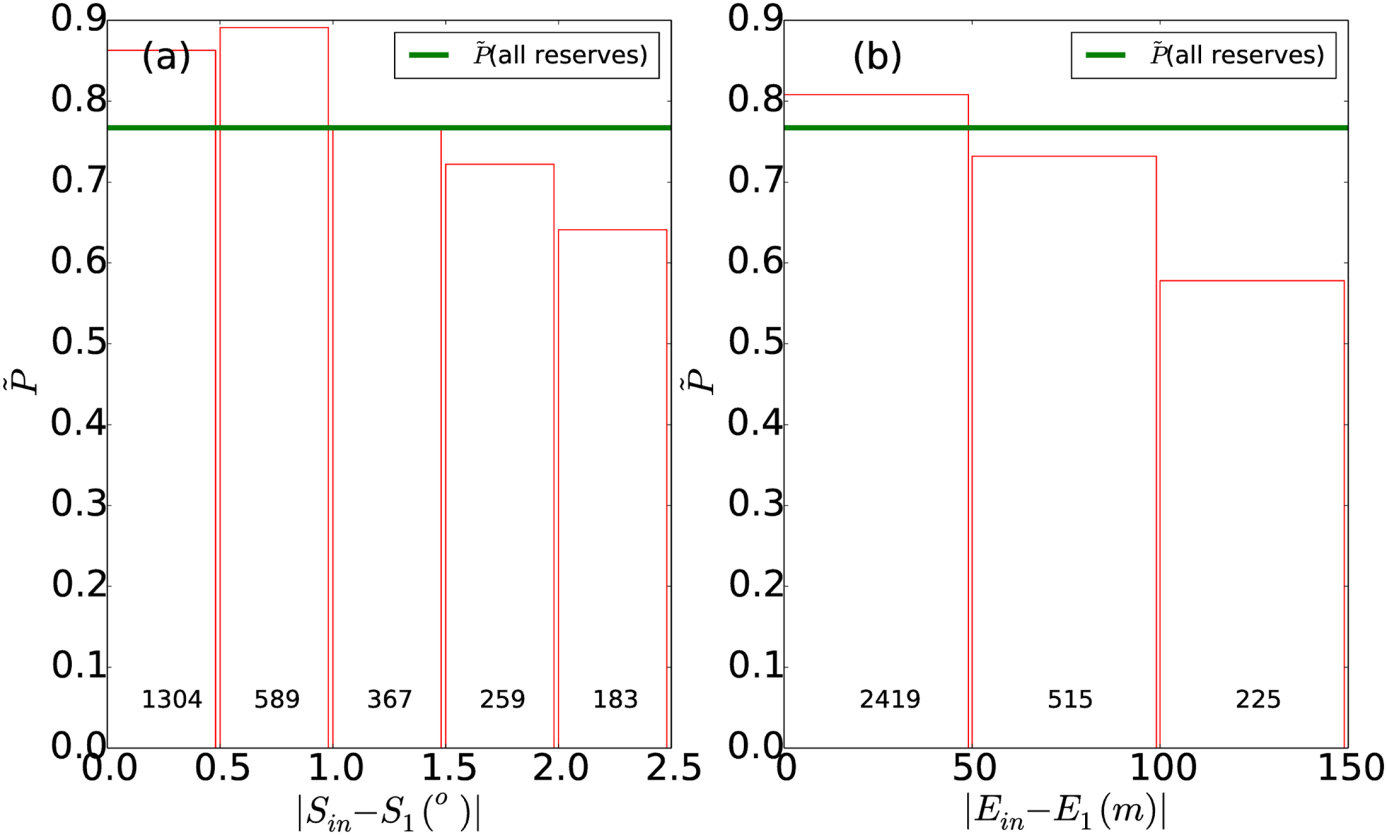
Effect on reserve performance of increasing difference in slope and elevation between inner and outer buffers. Median performance ($\tilde{P}$) of PAs as a function of difference in (a) slope (in degrees) and (b) elevation between inner 1 km and outer 1 km buffer zones. Numbers inside bars indicate number of reserves in each angle(elevation) range.

Fig 5 shows performance (*P*) of PAs, as a function of the difference in slope and elevation between inner and outer buffers. We found that effectiveness increased ($\tilde{P}$ decreased) when difference in slope between inner and outer buffers exceeded 1°. Across the subset of PAs where *S* in the inner buffer is within 1° of *S* in the outer 1 km buffer (|*S*_*in*_−*S*_1_| < 1°, n = 1893), $\tilde{P}=0.87$, significantly more (Kruskal-Wallis, *χ* ^2^ = 100.6, *p*<2e^−16^) than for reserves where the difference in slope is greater than 1° ($\tilde{P}=0.63$, n = 1483). Similar behavior was found for elevation, with $\tilde{P}=0.8$ for the subset of reserves where elevation was within 100 m (n = 2936), significantly more (K-W, *χ* ^2^ = 26.2, *p* = 3*e* ^−7^) than for reserves where the difference in elevation was greater than 100 m ($\tilde{P}=0.58$, n = 440). This suggests that reserves with steep slopes and high elevation are gaining substantial protection due to their geography. However, we find PAs still reduce forest loss rates after this effect has been removed. To distinguish between the effects of physical and legal protection, for the rest of the analysis we compare results for all PAs (n = 3376) against the subset of PAs with difference in slope of less than 1° and difference in mean elevation of less than 100 m between inner and outer buffers (n = 1804).

At the pan-tropical scale, 65% of all PAs reduced forest loss (that is P<1), with 49% of reserves classed as effective (defined as P<0.75), 33% as highly effective (P<0.5) and 19% as extremely effective (P<0.25). In the subset of PAs with similar slope and elevation the respective numbers were 60%, 41%, 27% and 14%. This confirms that a fraction of reserves are only effective because of their terrain. A meta-analysis of previous studies found a slightly better performance than we report here, with 82% of studies reporting lower rates of habitat loss within PAs compared to control areas [33]. [34] found that 40% of PAs experienced major management issues and were unlikely to deliver effective conservation. At the continental scale, we found that Australasia had the largest fraction of effective PAs (76%, 68% in subset), followed by the Neotropics (57%, 52% in subset), with Africa (48%, 43% in subset) and Asia (42%, 31% in subset) having fewer effective reserves (Table 1). In Asia the fraction of effective reserves is much lower in the subset of PAs with similar slope and elevation in inner and outer buffers. This suggests that PAs in Asia are less effective when there is no protection given by steep slopes and elevation.

At the pan-tropical scale, median performance ($\tilde{P}$) across all reserves was 0.77, suggesting PAs result in a median reduction in forest loss of 23%. Reserves with buffers of similar slope and elevation had $\tilde{P}$ of 0.87, suggesting a median reduction of forest loss of only 13%. This demonstrates that about 40% of the reduced forest loss across all PAs is due to terrain rather than legal status. Therefore PAs gain considerable ‘*de facto*’ protection from being preferentially sited on lands with steeper slopes and higher elevation than their surroundings. However, even after accounting for this effect, we find that PAs still confer some protection.

At the continental scale there was substantial variability in median performance ($\tilde{P}$). Across all PAs, better performing PAs (lower $\tilde{P}$) were found in Australasia (0.07) and the Neotropics (0.64) compared to Africa (0.78) and Asia (0.87). A consistent pattern was found for the subset of reserves, but with generally lower effectiveness (higher $\tilde{P}$) compared to all reserves. Across the subset of reserves, PAs in Australasia (0.16) and the Neotropics (0.72) were more effective compared to Africa (0.83) and Asia (1). In particular, performance of reserves in Asia is poor for the subset of reserves with similar slope and elevation in inner and outer buffers, demonstrating that the reduced deforestation in many Asian PAs is due to protection conferred by geography rather than legal protection. Median deforestation pressure (${\tilde{r}}_{out}$) was similar across the continents, except in Australasia where it is lower (Table 1), and as we show below does not explain variability in performance. More effective PAs in Australasia and Neotropics is consistent with a previous study [33].

**Fig 6:**
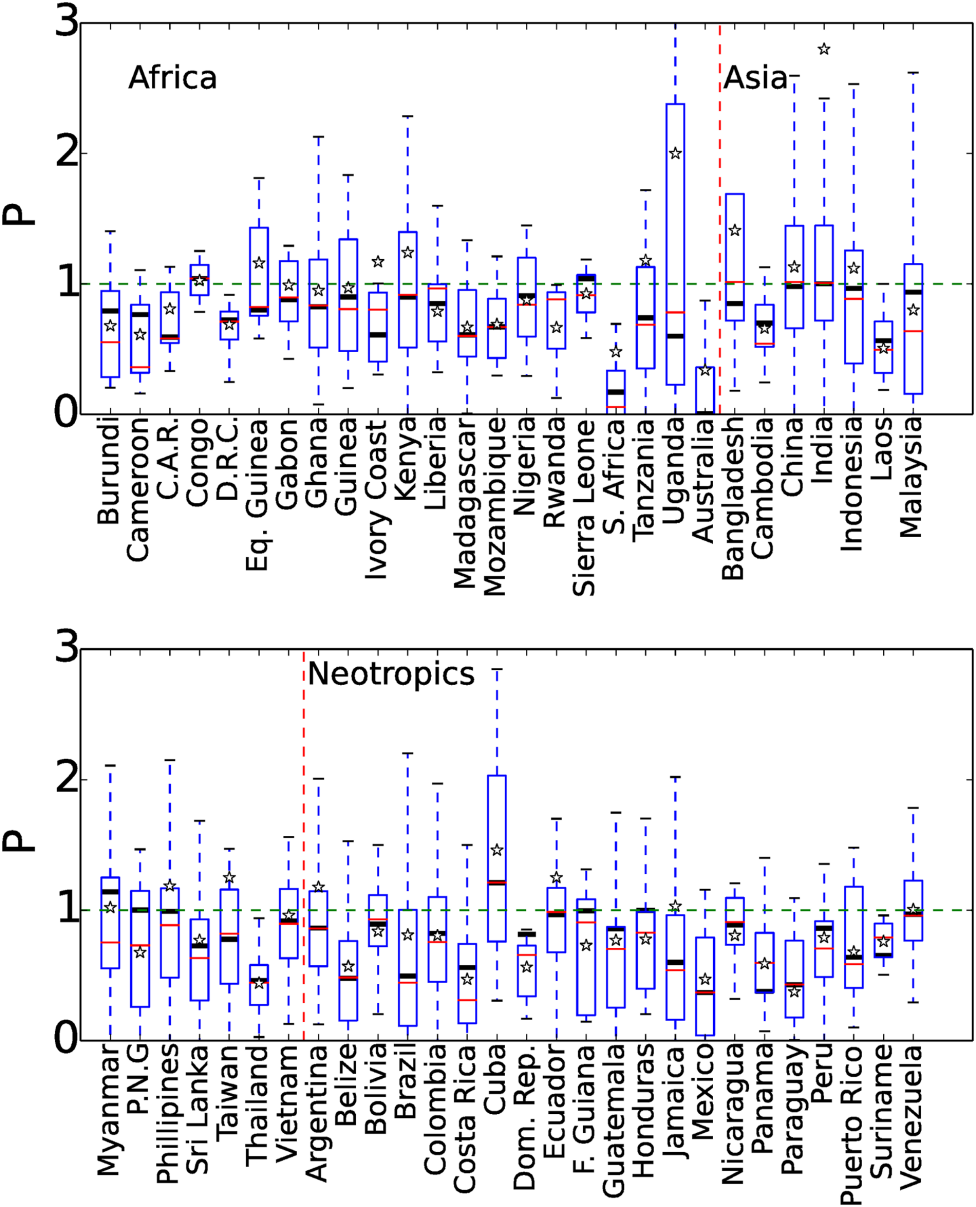
Variability of the performance (P) of PAs across the 56 analysed countries. Median performance (horizontal line) is shown for all PAs (red line) and for the subset of PAs with similar slope and elevation in inner and outer buffer zones (within 1° and 100 m respectively, thick black line). Box, whiskers and stars as for Fig 2.

**Fig 7:**
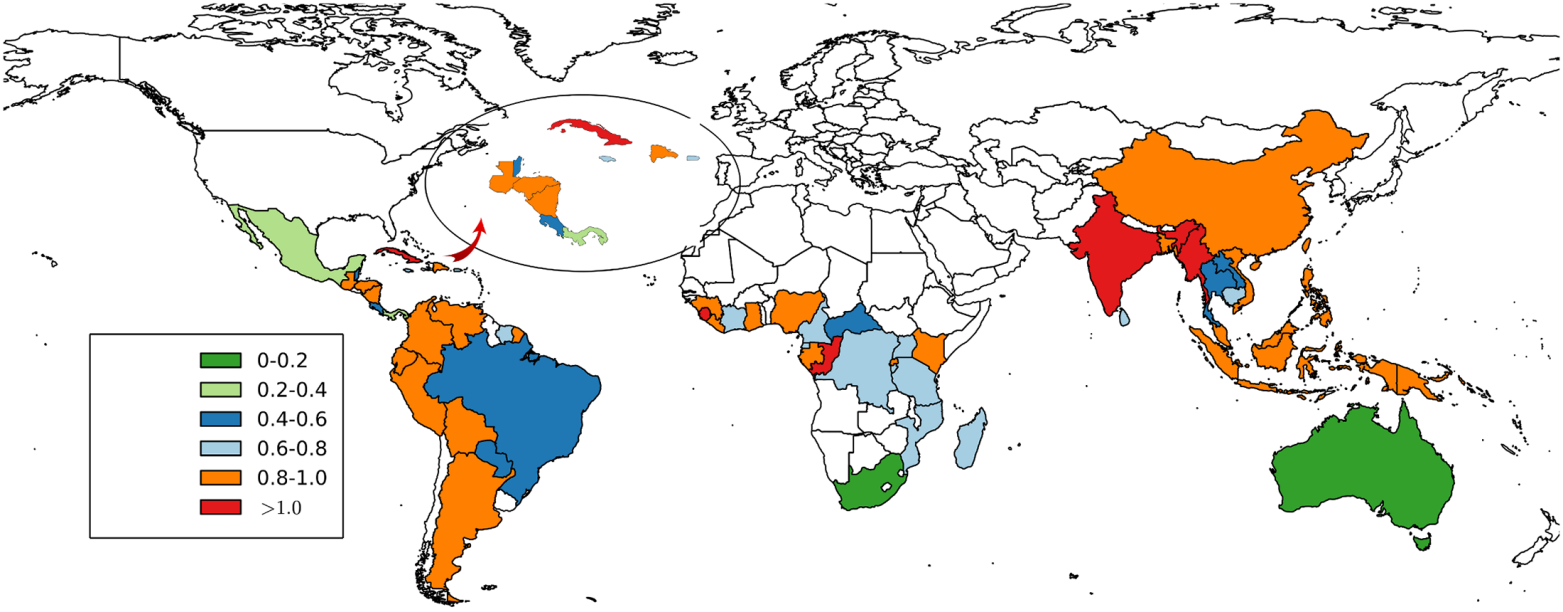
National median performance ($\tilde{P}$) of subset of PAs for the 56 countries in this analysis. P is calculated as *r*_*in*_/r_*inex*_, so P<1 indicates a country with effective PAs.

Fig 6 shows the variability in performance (*P*) across PAs within each of the countries we analysed. Median performance ($\tilde{P}$) is shown both for all reserves and for the subset of reserves with similar slope and elevation in inner and outer buffers. Fig 7 shows median performance($\tilde{P}$) for the subset of reserves across the 56 countries in this analysis. In China, India, Indonesia, Papua New Guinea, Philippines and Honduras the protective effect calculated across all reserves ($\tilde{P}<1$) is non-existent in the subset of PAs (*P* ≈ 1). This suggests that for these countries legal protection is ineffective, with forest loss only lower in PAs with higher elevations and steeper slopes than the surrounding region. Previous analysis has found weak management of PAs in Indonesia [5, 11, 17] and India [18]. Effectiveness was also substantially lower in the subset than for all reserves in Malaysia (0.94 compared to 0.6) and Costa Rica (0.56 compared to 0.3). The effectiveness of PAs in Laos and Thailand and in Belize and Costa Rica, contrasts with the relatively poor effectiveness across the rest of Asia and central America. Previous analysis found effective PAs in Costa Rica [19] and Brazil [13, 35]. [36] found PAs reduced forest loss in the Eastern Arc Mountains, Tanzania by 16–40%, matching $\tilde{P}=0.73$ in our work.

Global mean performance ($\bar{P}=0.96$) was greater than global $\tilde{P}$, due to variable performance of PAs with some performing very poorly (Fig 6). Of the 56 countries analysed we found that 54 countries (96%) had $\tilde{P}<1$ whereas only 38 countries (68%) had $\bar{P}<1$ (Fig 6), indicating that in some countries a minority of reserves were very ineffective. Our numbers are roughly consistent with [23], who found that PAs reduced forest loss in 75% of the 147 (including extratropical) countries they analysed.

To further explore the impact of the PA on forest loss rates, we analysed the relationship of forest loss rates within the reserve (r_*PA*_) and inner buffer (*r*_*in*_) with forest loss in the outer 1 km buffer (*r*_*out*_) and size of the PA (A, km^2^), for all reserves. We found consistent results if we complete the analysis on the subset of reserves. We found a significant relationship of forest loss rate within the PA (*r_*PA*_*) with forest loss in the outer buffer (*r*_*out*_) and reserve area (A), with *r_*PA*_* increasing with *r*_*out*_ and decreasing with A (*r*_*PA*_ = 0.825*r*_*out*_−0.0053*log*_10_(A) +0.004, *p* < 2*e* ^−16^, adjusted *R* ^2^ = 0.6). In contrast, forest loss rate within the inner buffer (*r*_*in*_) depended only on *r*_*out*_ with no significant dependence on A (*r*_*in*_ = 0.82*r*_*out*_, *p* < 2*e* ^−16^, adjusted *R* ^2^ = 0.6). The linear relationship between *r*_*out*_ and *r*_*in*_ indicates that deforestation pressure (*r*_*out*_) has minimal impact on reserve performance (*P* ≈ *r*_*in*_/*r*_*out*_). An exception is for the few PAs (n = 60) with very high deforestation pressure (*r*_*out*_>3.3%a^−1^) where the reserves were ineffective ($\tilde{P}=1$). For reserves with very little deforestation pressure (low *r*_*out*_), *P* may be > 1 simply because there is little deforestation to prevent. We found $\tilde{P}=1$ for those PAs (n = 154) where *r*_*out*_ < 0.01%*a* ^−1^.

We used multiple linear regression to explore the reasons for variability in median performance ($\tilde{P}$) at the national level. For all reserves, the best model featured national GDP/capita (p = 0.0004) and national rural population density (p = 0.05) (*F*_2,53_ = 8.41, *p* = 0.0007, adjusted *R* ^2^ = 0.212). Similar results were found when the subset of reserves was analysed but with the best model featuring only national GDP/capita (p = 0.0007) (*F*_1,53_ = 12.8, *p* = 0.0007, adjusted *R* ^2^ = 0.18). National GDP/capita appeared in the 20 best models for both datasets; with all reserves the third best-performing model was GDP/capita on its own (*F*_1,54_ = 8.41, *p* = 0.001, adjusted *R* ^2^ = 0.169). With both datasets (all and subset) increasing GDP/capita of a country improved predicted median reserve effectiveness (decreased $\tilde{P}$) by 0.17 for every U.S.$10000 increase in national GDP). However, the best models accounted for only about 20% of the variance in $\tilde{P}$, so there are important factors not accounted for here. In particular, we did not have data related to PA management (e.g., budgets, number or density of staff) which might affect performance. Furthermore, national statistics used in this analysis may not be representative of tropical forest regions.

## Conclusions

We analysed the effectiveness of 3376 protected areas (PAs) across the tropical moist forest biome using high-resolution global data of forest loss over the period 2000 to 2012. We compared forest loss within the entire PA, to forest loss in a 1 km buffer inside the PA and in multiple buffers up to 15 km outside the PA. We found small differences in forest loss between the outer buffers, with a larger difference in forest loss between the inner and outer buffer. The colocation of the PA boundary with the largest observed change in forest loss suggests that the change was due to the existence of the PA. We found no evidence for substantial displacement of deforestation to forest immediately outside PAs.

Despite a documented tendency for PAs to be located in remoter regions [21], nearly three quarters of the PAs we analysed experienced greater than 0.1% a^−1^ forest loss in their immediate surroundings (outer 1 km buffer), demonstrating widespread deforestation pressure in the tropics. Such extensive forest loss in regions surrounding PAs may result in forest fragmentation, with negative implications for ecological viability of the PAs [3, 37]. We also found substantial rates of forest loss within PAs, with a rate of pan-tropical forest loss within PAs of 0.13% a^−1^. Fastest rates of PA forest loss occurred in Central America, West Africa and parts of Asia (>0.3% a^−1^), with slower rates in the Amazon, Congo and Australia. At the continental scale forest loss within PAs was greatest in Asia (0.25% a^−1^), and less in Africa (0.1% a^−1^), Neotropics (0.1% a^−1^) and Australasia (0.03% a^−1^). At the global scale 39% of PAs experienced forest loss greater than 0.2% a^−1^.

We assessed the effectiveness of PAs through comparing forest loss in the inner and outer 1 km buffers. Comparison of forest loss rates within these adjacent buffers partially removes issues caused by locational bias of PAs. To remove potential bias introduced by differences in terrain, we also analysed a subset of PAs (n = 1804) with similar slope (within 1°) and elevation (within 100 m) in inner and outer 1 km buffers. At the pan-tropical scale, we found 65% (60%) of all PAs (subset) had lower forest loss in the inner 1 km buffer than the outer 1km buffer(corrected for distance effects). Within the subset of reserves, only 41% of PAs reduced deforestation by at least 25%. We defined performance (*P*) of a PA as the ratio of forest loss in the inner 1 km buffer compared to the loss that would have occurred in the absence of the PA, calculated as the forest loss rate in the outer buffer corrected for any difference in deforestation pressure between inner and outer buffers. Median performance ($\tilde{P}$) was 0.77 (0.87 for subset), meaning a reduction in forest loss within PA of 23% (13%). Terrain therefore accounted for nearly half of the reduced forest loss within PAs but with substantial variability from country to country.

We found that Australia, South Africa, Mexico, Panama and Thailand have the best performing PAs. French Guiana, Suriname and Cameroon have very low rates of deforestation (*r*_*out*_ < 0.05% a^−1^) both within and outside PAs. Cuba, Myanmar and Sierra Leone ($\tilde{P}>1$) as well as Indonesia, China, Honduras, India, Papua New Guinea, Venezuela and the Philippines ($\tilde{P}\approx 1$ for subset of reserves) have the worst performing PAs.

We found that PAs were most effective in Australasia (Australia and Papua New Guinea; $\tilde{P}$ = 0.16) and less effective in the Neotropics ($\tilde{P}=0.72$) and Africa ($\tilde{P}=0.83$). In Asia, performance of PAs was particularly poor with the median performing PA providing no legal protection against forest loss ($\tilde{P}=1.0$). However, comparisons at the continental level ignore important variability. For example, PAs in Thailand and Laos were found to be very effective. At the national level the effectiveness of PAs improved with increased GDP per capita and declined with increased rural population. Continued rural population growth [38, 39] may therefore threaten the future effectiveness of PAs. Increased effectiveness of PAs with increased GDP per capita may be due to increased financial support of PAs within these countries. This suggestion requires further investigation.

Overall, we found that PAs do reduce deforestation but with substantial variability within and between countries. Future work to better understand the reasons for this variability in the performance is needed. Our buffer zone analysis may overestimate effectiveness of PAs [16, 19–21], suggesting our results may provide an upper estimate for the effectiveness of PAs in reducing forest loss. In any case, our work demonstrates there is substantial potential for improved management and reduced forest loss within many PAs. The low effectiveness of PAs across many countries in Asia is worrying given the fast rates of forest loss within this region. We note that our paper only measures reserve performance in terms of preventing deforestation. Other issues such as hunting, grazing or the selective logging of valuable trees is not addressed. Nonetheless, our work highlights the crucial need for increased political and financial support, and robust monitoring, of tropical forest PAs [4].

## Supporting Information

S1 Dataset Deforestation rates for all analysed reserves. (XLS)
